# Preface to ‘Shortcuts to adiabaticity: theoretical, experimental and interdisciplinary perspectives’

**DOI:** 10.1098/rsta.2021.0284

**Published:** 2022-12-26

**Authors:** Mikio Nakahara, Xi Chen, Yue Ban, Shumpei Masuda

**Affiliations:** ^1^ Research Institute for Science and Engineering, Kindai University, Higashi-Osaka 577-8502, Japan; ^2^ Department of Physical Chemistry, University of the Basque Country UPV/EHU, Apartado 644, 48080 Bilbao, Spain; ^3^ EHU Quantum Center, University of the Basque Country UPV/EHU, 48940 Leioa, Spain; ^4^ TECNALIA, Basque Research and Technology Alliance (BRTA), Parque Científico y Tecnológico de Bizkaia, Astondo Bidea,Edificio 700, 48160 Derio, Spain; ^5^ Research Center for Emerging Computing Technologies, National Institute of Advanced Industrial Science and Technology (AIST),1-1-1 Umezono, Tsukuba 305-8568, Japan

Continued advances in understanding and precise manipulation of quantum properties as they appear in the microscopic world are paving the way for a ‘second quantum revolution’. A novel scenario of quantum technologies, which includes quantum information, quantum sensing and metrology, quantum computation, quantum communication, etc., is under development worldwide. These novel technologies exploit quantum properties in existing systems, such as superposition and entanglement, for the development of unprecedented tools and devices. A prominent example is the quantum computer—a new paradigm of computation—relying on the precise addressing of quantum bits (qubits), which is expected to accelerate tasks such as material design, machine learning, computational chemistry, drug design, cryptography and financial modelling among others and may dramatically change our lives in the near future.

Precise control of quantum systems in modern platforms is a typical requirement for realizations of a quantum computer in the presence of decoherence, which is a degradation of a quantum state due to interaction with its environment. Adiabatic quantum control is known to attain high precision but it requires a long execution time to avoid non-adiabatic transitions. However, such slow control is not desirable in view of decoherence. Although quantum error-correcting codes and topological quantum computing have been proposed as ways to suppress or overcome decoherence or errors, these strategies require additional qubits for encoding or topological systems that support braiding of non-Abelian anyons, which have not yet been physically realized.

Shortcuts to adiabaticity (STA), originally put forward to speed up the adiabatic process, have become a powerful technique for quantum control in a fast, accurate and robust way with respect to environmental decoherence. With the current state of the art in different quantum platforms, e.g. superconducting qubits, spin qubits, trapped-ion qubits and cold atoms, the external fields or time-varying interactions are designed to achieve the final state of the adiabatic time-evolution in a shorter time, even though the intermediate state involves transitions among instantaneous eigenstates of the modified Hamiltonian in general. STA are broadly divided into four main categories: dynamical invariant formalism, counterdiabatic (or transitionless) quantum driving, fast-forward scaling theory and quantum brachistochrones. Furthermore, STA and their variants can be further combined with optimal control theory and machine learning, in order to achieve both robustness and optimality. STA can increase the fidelity of gate operations and state preparation without using extra qubits, and can even reduce the number of qubits and quantum gates, toward the quantum advantage in the noisy intermediate-scale quantum computer era.

STA have a wide range of applications not limited to quantum control and quantum computation, even beyond physics. Despite this recent surge of STA research, the subject has not yet become textbook material, and although there are a few review papers and special issues of journals on STA, beginners with a reasonable background in quantum physics still face difficulties in starting research in this fascinating field. This theme issue aims to fill the gap between standard quantum mechanics textbooks and state-of-the-art research papers, by first reviewing the fundamentals of STA by its experts and founders, and then presenting recent work, both theoretical and experimental, by scientists at the forefront of the field. STA have become a burgeoning field of quantum physics, with more applications spanning from physical chemistry, statistic physics and, more recently, to biophysics. Examples include, but are not limited to, classical mechanical system, classical biological processes and evolutionary rates in biology. From different perspectives, STA can speed up the operation of a quantum perceptron, a building block of a quantum neural network, which is expected to be a useful tool for quantum computing and quantum machine learning. An equally unexpected connection between STA and the Lax pair formalism of completely integrable systems has been discovered recently. A digitized counterdiabatic quantum approximate optimization algorithm is applied to improve classical optimization problems where it decreases the circuit depth, reduces the number of optimization parameters and provides a better approximate trial state which is beneficial for finding the optimal target state. All these implications illustrate well the interdisciplinary nature of the subject. In view of this, authors from other scientific disciplines, who found unexpected applications of STA, are also invited to contribute.

We believe that this theme issue is very timely and will attract readers from many scientific communities. We also hope that the contents of this theme issue will enable young researchers to begin their research of STA and find the future direction of STA research in their own fields.

## Data Availability

This article has no additional data.

